# The Treatment of True Membranous Croup

**Published:** 1875-11

**Authors:** W. H. Vail

**Affiliations:** Cornwall-on-Hudson, N. Y.


### Selections.

---

### THE TREATMENT OF TRUE MEMBRANOUS CROUP.

BY W. H. VAIL, M.D., CORNWALL-ON-HUDSON, N. Y.

My experience during the last spring, with four very severe cases of unmistakable membranous croup, has led me to consider it a disease as amenable to treatment as dysentery or remittent fever. The symptoms in all the cases being so similar, and coinciding so exactly with those attributed in Dr. Flint's practice to laryngitis with exudation of lymph, I will not detain you with the recital of them—only referring to them as occasion may require in connection with the treatment. Upon seeing a patient suffering from this disease, I immediately give him a full dose of calomel, from fifteen to thirty grains, to be repeated in six hours if the bowels have not operated. It allays the fever and acts kindly on all the secretions. When this effect is desired in any disease, large doses must be given, according to Dr. Lente's plan. All of the popular hue and cry against this valuable medicine, and all of our bad results and disappointments from its use, have come from our giving it in too minute doses. It matters very little how much you give, if you only give enough to operate on the bowels, or follow it with something which will assist it to operate. At the same time, I order the patient to be kept day and night in the kitchen, or a room which can as readily be kept heated at a temperature of 90° Fahrenheit, and the air loaded with moisture until it runs down the windows as we see it on washing-days. To make this measure effective, Dr. Sayre's plan must be followed, of keeping the temperature at 90° or 95° if necessary, as I found it in one case. You will be surprised and gratified in a few hours at finding your patient breathing easily and sleeping quietly, to whom, before, each breath was a struggle, and there was no such thing as sleep. You must impress upon the friends the importance of maintaining the heat and moisture. If the thermometer falls to 80° or 75°, the old trouble will return. In one case, I kept the heat at 90° for three days and two nights before it seemed safe to let it subside.

These two remedies (calomel, and heat and moisture,) seem to stop at once the further formation of the membrane, to loosen what is already formed, or cause it to be absorbed. They acted like a charm with three of my little patients; the fourth was moribund when I first saw him, and died in less than twenty-four hours, asphyxiated. No attempt at tracheotomy was made in his case, as the membrane evidently extended to the bronchioles, and no operation could relieve him.

To cure any sore throat that may exist, I dissolve half a drachm of chlorate of potash in a glass of water, and give a teaspoonful every two hours. I give it in all cases, for it is a tonic, and acts well on the membrane, wherever it may be.

To maintain the strength, I rely on quinine, and I find the following simple prescription the most palatable and acceptable to the stomach:

℞.—Cinco. quiniæ, . . . . . . ʒ ss.

Syr. simplicis, . . . . . . ℥ ij.

M. Pulverize and triturate thoroughly. From a half to one teaspoonful every four hours.

Of course, milk, eggs, milk-punch, beef-tea, lemonade, flaxseed-tea, etc., enter as nourishment into the treatment—to be varied as the strength and taste of the little ones may require.

That these were cases of true croup, as distinguished from spasmodic croup, there is no doubt in the mind of the writer, and is proved by their symptoms not being those of any other disease, and their clinical history corresponding with what the best authors teach us to look for in that affection. The membrane was visible on the tonsils in two cases, and existed, I believe, in the larynx and trachea of the remaining two, but did not happen to extend to the throat, or was prevented from extending by the remedies used. Though I watched, and asked the friends to watch, for anything in the shape of membrane that might be coughed up, nothing of the kind was seen. Whether the membrane was swallowed, or dissolved or absorbed by the remedies, I do not pretend to say.

As to sequelæ, one was unable to speak above a whisper for a fortnight, and another is now gradually recovering his speech after a lapse of nearly two months. No medicine was given to cut short this sequel, time alone being relied on. No originality as to remedies is claimed by the writer. He only hopes others may be gratified with the good results which followed his adoption of this plan of treatment, and that it may prove a means of reducing the fatality of this so fatal disease. If I meet with further success in this direction, I shall certainly report it, and hope others will also. *New York Medical Journal.*

